# Supplementary figures and images for: Monitoring of UN sustainable development goal SDG-9.1.1: study of Algerian “Belt and Road” expressways constructed by China
Source: PeerJ. 2020 Jun 2;8:e8953. doi: 10.7717/peerj.8953 (PMC7274168; doi:10.7717/peerj.8953)

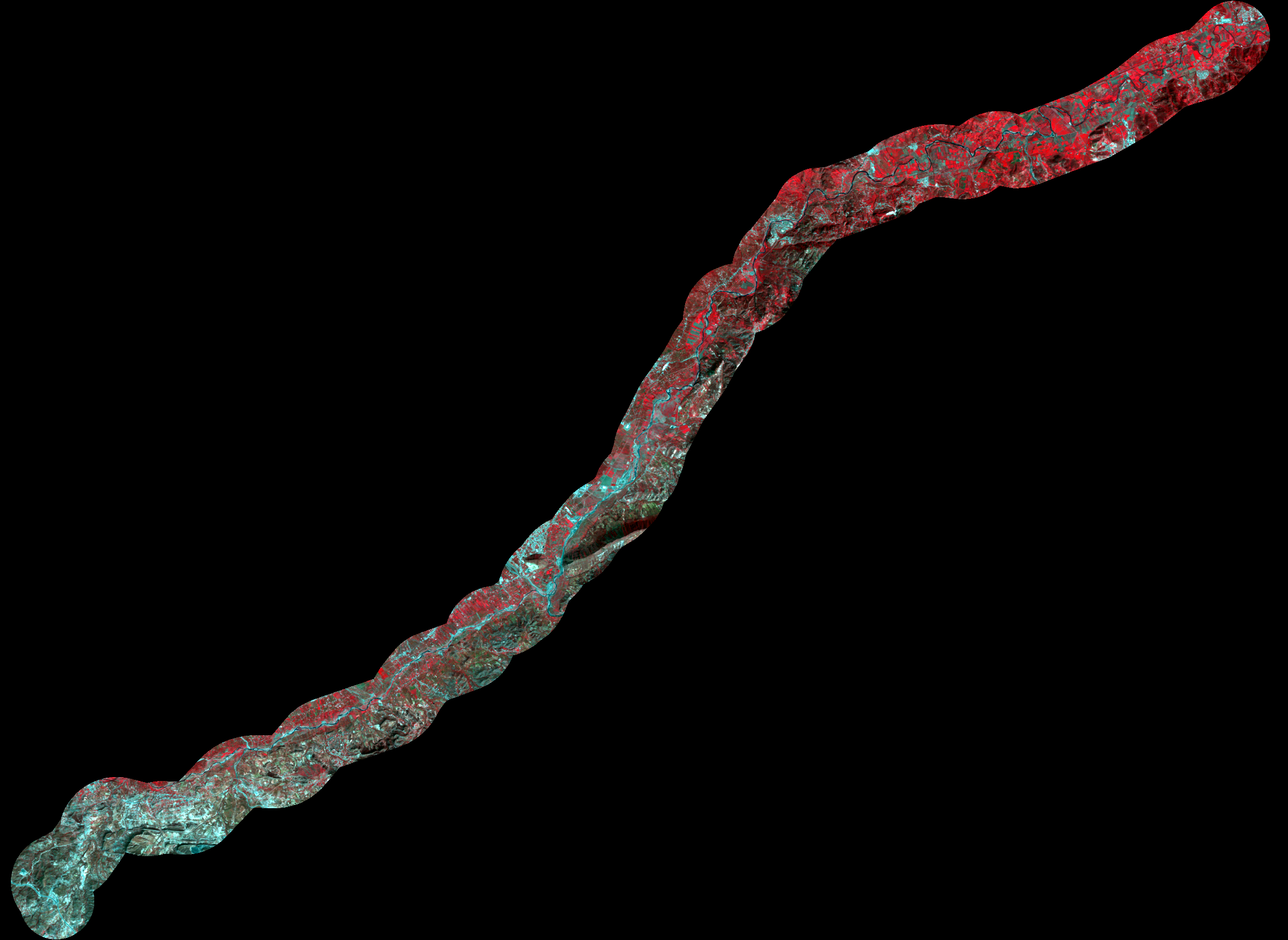

Supplement: Supplemental Information 1 [file peerj-08-8953-s001.zip › 2011 Landsat image.tif]

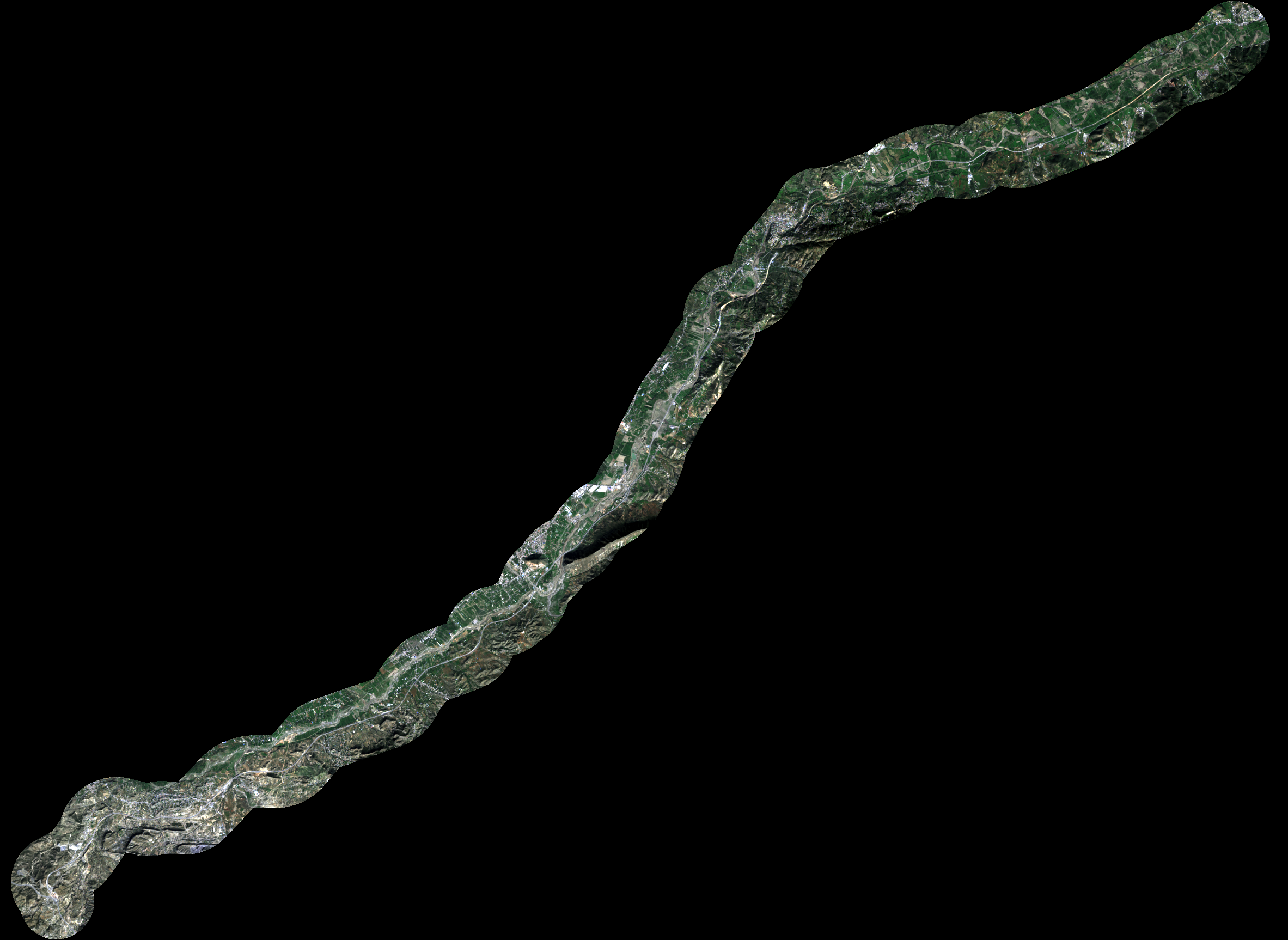

Supplement: Supplemental Information 3 [file peerj-08-8953-s003.zip › 2019 Landsat image.tif]

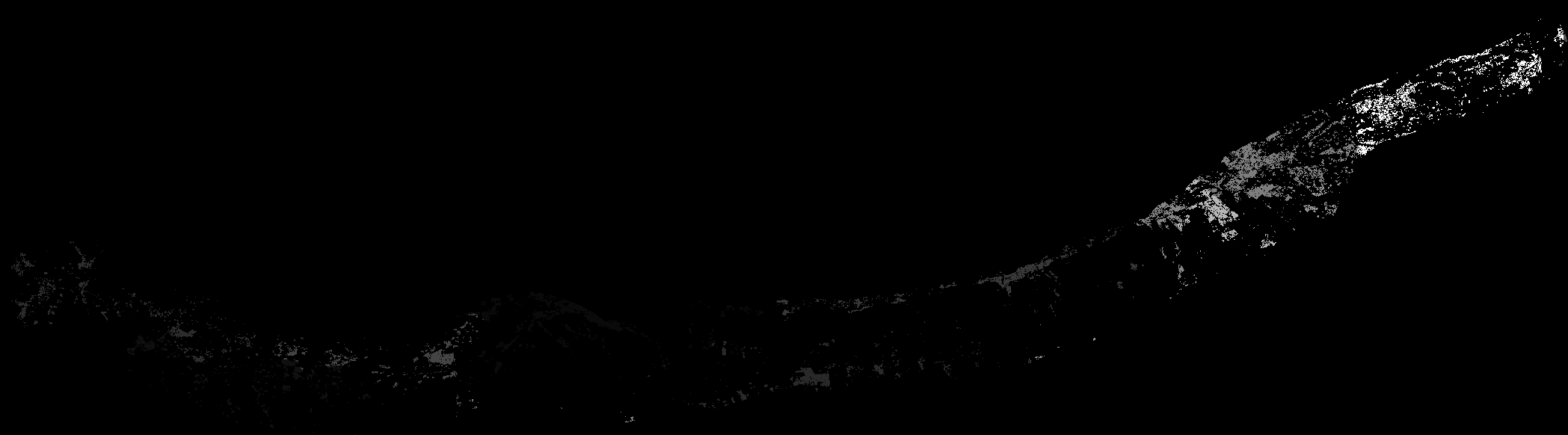

Supplement: Supplemental Information 9 [file peerj-08-8953-s009.zip › 2015 hrsl Population Image.tif]
